# Prehistoric Decay of the Teeth

**Published:** 1887-01

**Authors:** C. T. Terry


### PREHISTORIC DECAY OF THE TEETH.

BY DR. C. T. TERRY.

Read before the American Dental Society of Europe, Aug. 2, 1886.

What is meant by the prehistoric decay of teeth spoken of by those who have written on this subject ? Do they mean to imply the condition of the teeth of people in general in prehistoric times, or do they mean the condition of the teeth of that small class of prehistoric inhabitants of the earth of which there is some record, not written, but handed down to us in the form of human remains found in tombs ?

To what class of the prehistoric inhabitants do these remains belong ? Certainly not to that great order who were nothing more than slaves; people who could not live otherwise than in the simplest manner, and subsist upon the simplest food of that period.

What was the manner of life of those whose skeletons have been preserved ? Having but little knowledge of hygienic laws, and as little use for such knowledge, being at the same time the privileged class, how would they probably live ? If the rich in our times, with their knowledge of sanitary matters, indulge in luxury to an unlimited extent, what could be expected of that small class who ruled supreme in prehistoric times ? Fortunately for them, as regards health, they could not indulge in the same number of preparations for the stomach in the way of food and drink that we can, but in other forms of dissipation this class, no doubt, had advantages over us. For thousands of years the ruling class have habituated themselves to the use of fermented drinks, especially wine, drinking to excess new wine, thereby producing derangements in the functions of the stomach.

Among the excavations at Pompeii the immense earthen vessels for holding wine are conspicuous, and there are many other evidences of great profligacy among the inhabitants of this ancient city. I examined several skulls of those who were buried in the ashes, and found many instances of decay in the teeth. I think it can be proved that the ruling classes of Europe have not lived hygienically for hundreds of years. The skeletons which have been preserved generally belong to this class, for they were found buried in churches or tombs that have been kept in good condition for centuries. How many of the skeletons of simple peasants, who lived at the same period, have been preserved ?

In order to ascertain the normal condition of the teeth of the human race, it is necessary to go back to the period when they chose their food instinctively. There was a time, no doubt, when man had no more inventive ability than the monkey. But man, of all animals, is most progressive. When he had reached that period of mental development which would enable him to invent, what would be his first efforts in this direction ? Naturally, in his uncivilized condition, he would bring his creative ability to bear upon something that had to do with his food, or upon some weapon of defense or of destruction. Probably the first change which he made in his food was to cook it, and from this he has gradually gone on until our diet has reached its present complicated or artificial condition. Perhaps his very first change, even before cooking his food, was to construct a habitation of some kind for protection against heat and bad weather.

The more he advanced intellectually, the more complicated and artificial became his manner of living. No doubt it was several generations, or even hundreds of years after man began to change from his normal condition, before decay of his teeth began, on account of the comparatively primitive way in which he was still compelled to live, and the perfectly healthy condition of his osseous system. Undoubtedly, before man began to change his normal condition, when he was compelled to choose his food instinctively, there was not such a thing known as a decayed tooth. If so, it was the result of accident, such as fracture in battle or otherwise, forming a receptacle for a quantity of food which fermented and dissolved the bone; and it is still a question, even under such circumstances, if the healthy condition of the fluids of his mouth would not have prevented decay in such a case.

As man advanced in civilization, his habitation became more luxurious, his food and drink more complicated, and his occupations less invigorating, and confinement of the young under shelter for purposes of education more frequent. This gradual departure or change from the normal condition of the human race, which has been going on for thousands of years, has finally caused a great degeneration in the tissues and functions of the system, bringing the teeth into a condition to be acted upon in the mouth by direct causes of decay, such as acids and putrefaction. I am quite sure that agents which cause decay of teeth in the mouths of the present civilized generation would be harmless in the mouth of the uncivilized man who chooses his food instinctively.

Decay of the teeth is certainly not of modern origin. I believe it to have existed to a certain extent for hundreds of years. It has been increasing gradually, and has kept pace with the rapid increase of inventions in general. There has been about as much inventive ability brought to bear upon that which enters the stomach of the civilized human being in the way of food, drink, medicine, etc., as upon machinery. What wonderful strides we have made in mechanical and physical discoveries and inventions during the last two hundred years; and what a vast accumulation of mixtures have been invented for the human stomach in the same time.

How much more confinement in impure air in schools, factories, etc., is there than formerly, and how much less pure air we inhale than did our uncivilized ancestors. If the inhabitants of this world at a former period were as far advanced as we now are in civilization, I am also sure that their teeth were as bad as ours at the present time, and there is no doubt if, at any time past, man had reached the inventive perfection which we have, he would have lived just as artificially as we do now, the result of which is decay of the the human teeth, notwithstanding all our scientific knowledge, which at best can only prevent decay to a certain extent; and unless we can discovor a manner of living which will again produce the perfectly healthy organisms of man in his normal condition, when choosing his food instinctively, teeth will continue to retrograde.

